# Insight into the Clonal Lineage and Antimicrobial Resistance of *Staphylococcus aureus* from Vascular Access Infections before and during the COVID-19 Pandemic

**DOI:** 10.3390/antibiotics12061070

**Published:** 2023-06-18

**Authors:** Chih-Chen Kao, Chi-Hsiang Lai, Min-Yi Wong, Tsung-Yu Huang, Yuan-Hsi Tseng, Chu-Hsueh Lu, Chien-Chao Lin, Yao-Kuang Huang

**Affiliations:** 1 Division of Thoracic and Cardiovascular Surgery, Chiayi Chang Gung Memorial Hospital, Chiayi 10020, Taiwan; mdtobe@cgmh.org.tw (C.-C.K.); kathy8512@cgmh.org.tw (C.-H.L.); mynyy001@cgmh.org.tw (M.-Y.W.); gsl3639000@cgmh.org.tw (C.-H.L.); m7018@cgmh.org.tw (C.-C.L.); 2 College of Medicine, Chang Gung University, Taoyuan 33041, Taiwan; p122273@cgmh.org.tw; 3 Division of Thoracic and Cardiovascular Surgery, Chiayi Hospital, MOHW, Chiayi City 10020, Taiwan; 4 Division of Infectious Diseases, Department of Internal Medicine, Chang Gung Memorial Hospital, Chiayi 10020, Taiwan; 8802003@cgmh.org.tw; 5 Division of Cardiovascular Surgery, New Taipei Municipal Tu-Cheng Hospital, New Taipei City 23656, Taiwan

**Keywords:** vascular access infections (VAIs), *Staphylococcus aureus*, antibiotic resistance, antibiotic resistance genes, multilocus sequence typing, molecular characterization

## Abstract

Patients receiving hemodialysis are at risk of vascular access infections (VAIs) and are particularly vulnerable to the opportunistic pathogen *Staphylococcus aureus*. Hemodialysis patients were also at increased risk of infection during the COVID-19 pandemic. Therefore, this study determined the change in the molecular and antibiotic resistance profiles of *S. aureus* isolates from VAIs during the pandemic compared with before. A total of 102 *S. aureus* isolates were collected from VAIs between November 2013 and December 2021. Before the pandemic, 69 isolates were collected, 58%, 39.1%, and 2.9% from arteriovenous grafts (AVGs), tunneled cuffed catheters (TCCs), and arteriovenous fistulas (AVFs), respectively. The prevalence of AVG and TCC isolates changed to 39.4% and 60.6%, respectively, of the 33 isolates during the pandemic. Sequence type (ST)59 was the predominant clone in TCC methicillin-resistant *S. aureus* (MRSA) and AVG-MRSA before the pandemic, whereas the predominant clone was ST8 in AVG-MRSA during the pandemic. ST59 carrying the *ermB* gene was resistant to clindamycin and erythromycin. By contrast, ST8 carrying the *msrA* gene was exclusively resistant to erythromycin. The ST distribution for different VAIs changed from before to during the pandemic. The change in antibiotic resistance rate for different VAIs was closely related to the distribution of specific STs.

## 1. Introduction

*Staphylococcus aureus* has been a key opportunistic pathogen in humans. It can cause various infections and was first discovered as part of the staphylococcal disease in 1880 by surgeon Alexander Ogston in pus from a surgical abscess [1]. Approximately 40–60% of the human population is intermittently colonized by *S. aureus*, and approximately 20% is persistently colonized [2]. Individuals colonized with *S. aureus* are at increased risk of infection; the rate of colonization is higher in those who inject drugs or have type 1 diabetes, d*ermA*tologic conditions, immunodeficiency syndrome, or hemodialysis than in the general population [3,4]. After the first appearance of methicillin-resistant *S. aureus* (MRSA) in 1961, shortly after methicillin was introduced, MRSA spread globally. It was first documented in Taiwan in the early 1980s and spread rapidly in the 1990s [5]. In the past two decades, a decline has been observed in healthcare-associated MRSA, and community-acquired MRSA has increased in incidence [6]. ST239 and ST59 are the major clones of *S. aureus* in Taiwan. ST239 is healthcare-associated, whereas ST59 is community-associated. The reduction in ST239 and increase in ST59 in hospital settings since the 2010s indicates the effective adaptation of ST59 to hospital environments. ST59 appears to be a nosocomial clone capable of causing invasive infection [7,8].

Infections are the second most common cause of hospitalization, morbidity, and mortality in hemodialysis patients after cardiovascular events. This population has a higher risk of invasive *S. aureus* infection than the nondialysis population [9]. *S. aureus* carriers on hemodialysis have a 1.8- to 4.7-fold higher risk of vascular access infections (VAIs) and bacteremia compared with noncarriers [10]. The risk of MRSA infection in hemodialysis patients is 100 times more than the general population [11]. Patients receiving hemodialysis are highly susceptible to VAIs because of their long-term necessity for vascular access, frequent puncture of vascular access sites, repeated hospitalization, frequent and long-term use of antibiotics, and immunosuppression [12]. Vascular access type is associated with the risk of infection; the common vascular access types are tunneled cuffed catheters (TCCs), arteriovenous grafts (AVGs), and arteriovenous fistulas (AVFs), in order of decreasing infection risk [13]. Although AVFs have the benefit of a high patency rate and low infection rate, they have the disadvantage of a high primary failure rate because of early thrombosis and failure to mature, which partly contributes to the higher incidence of catheter use.

Ongoing molecular surveillance is essential for preventing *S. aureus* infection in healthcare facilities. During the coronavirus disease 2019 (COVID-19) pandemic, numerous infection control and prevention measures were implemented in response to COVID-19, and these measures brought additional benefits in reducing other infections [14]. However, patients receiving hemodialysis are especially vulnerable to COVID-19 because of their greater comorbidities and frequent healthcare visits, which are often made using public transportation even under pandemic conditions, further exposing them to the risk of community-transmitted infection. Therefore, this study examined the molecular epidemiology and antibiotic resistance of *S. aureus* isolates obtained from VAIs before versus during the pandemic and clarified the correlation between access types, genetic background, and antibiotic resistance.

## 2. Results

### 2.1. Distribution of Isolates from Different VAI Types

**Figure 1:**
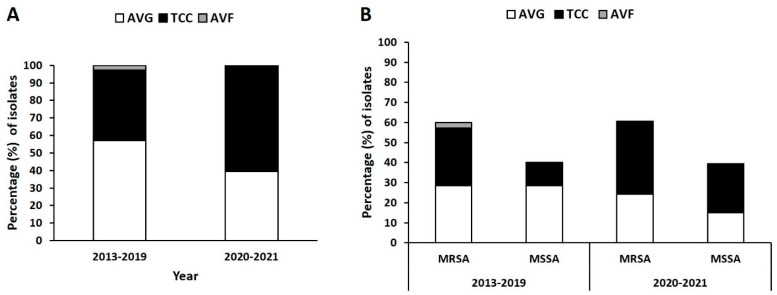
Distribution of *Staphylococcus aureus* isolates from vascular access infections (VAIs) before and during the COVID-19 pandemic (2013–2019 vs. 2020–2021). Classified for (**A**) two periods and (**B**) methicillin-resistant *S. aureus* (MRSA) and methicillin-sensitive *S. aureus* (MSSA).

In total, 102 isolates were collected from three types of VAI: AVGs (*n* = 53), TCCs (*n* = 47), and AVFs (*n* = 2); 69 isolates were collected before the COVID-19 pandemic (November 2013–November 2019), and 33 isolates were collected during the pandemic (March 2020–December 2021). As depicted in Figure 1A, the prevalence of AVG, TCC, and AVF infections in the prepandemic period were 58%, 39.1%, and 2.9%, respectively. During the pandemic, the prevalence of AVG infection was lower at 39.4%, whereas TCC infection was higher at 60.6%.

The prevalence of MRSA and methicillin-sensitive *S. aureus* (MSSA) from AVG infection were both 29% before the pandemic but lower at 24.2% and 15.2% during the pandemic, respectively (Figure 1B). The ratio of AVG-MRSA to AVG-MSSA during the pandemic was 1.6:1 compared with 1:1 before the pandemic, indicating an increase in MRSA in the AVG infection population during the pandemic. By contrast, the prevalence of MRSA and MSSA from TCC infection was 27.5% and 11.6%, respectively, in the prepandemic period, and 36.4% and 24.2% during the pandemic, respectively. The ratio of TCC-MRSA to TCC-MSSA was 1.5:1 during the pandemic period compared with 2.4:1 before the pandemic, indicating a decrease in MRSA in the TCC infection population.

### 2.2. Distribution of SCCmec Type in MRSA Isolates

Of the 102 *S. aureus* isolates, 61 were identified as MRSA, of which 60 were *mecA*-positive MRSA and 1 was oxacillin-resistant *mecA*-negative MRSA.

Of the 61 MRSA isolates, the predominant SCC*mec* type was SCC*mec* type IV, followed by SCC*mec* V. The prevalence of SCC*mec* type IV was approximately 50% in both periods, whereas the prevalence of type V increased from 19.5% before the pandemic to 30% during it. Conversely, SCC*mec* III decreased from 17.1% to 10%. SCC*mec* IV and V are present mainly in community-associated MRSA isolates, indicating that VAI isolates tend to be community-associated. The most prevalent SCC*mec* type of AVG-MRSA was SCC*mec* IV, with a prevalence of 65% before the pandemic that increased to 75% during the pandemic. By contrast, TCC-MRSA predominantly carried SCC*mec* III and V elements before the pandemic, whereas SCC*mec* IV overtook III to become the second predominant type after SCC*mec* V during the pandemic.

### 2.3. Distribution of Sequence Types (STs) in VAI Isolates

**Figure 2:**
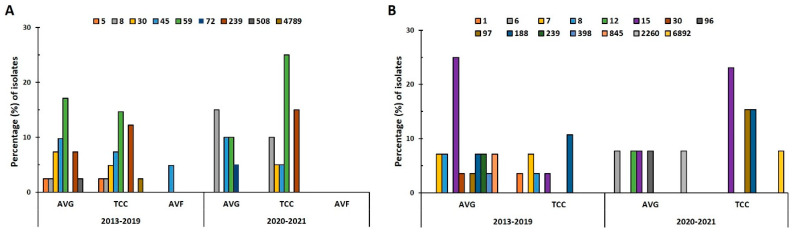
Distribution of sequence types (STs) in *S. aureus* isolates before and during the pandemic (2013–2019 vs. 2020–2021). Prevalence of STs in all (**A**) methicillin-resistant *S. aureus* (MRSA) and (**B**) methicillin-sensitive *S. aureus* (MSSA) isolates from different VAIs in the two periods.

In the prepandemic period, 8 STs were identified in 41 MRSA isolates, and the predominant ST types were ST59 (31.7%, 13/41), ST45 (22%, 9/41), ST239 (19.5%, 8/41), and ST30 (12.2%, 5/41; Figure 2A). During the pandemic, only 6 STs were identified in 20 MRSA isolates, and the predominant STs were ST59 (35%, 7/20) and ST8 (25%, 5/20). ST59 was the predominant ST in both periods, with its prevalence being slightly higher during the pandemic; AVG-MRSA ST59 (17.1%) was slightly more prevalent than TCC-MRSA ST59 (14.6%) before the pandemic, whereas TCC-MRSA ST59 (25%) increased remarkably during the pandemic. AVG-MRSA ST30 and ST45 were slightly more prevalent than TCC-MRSA before the pandemic. ST45 levels remained consistent during the pandemic; however, no AVG-MRSA ST30 isolates were detected. ST8 prevalence in AVG-MRSA and TCC-MRSA increased substantially during the pandemic, and ST8 became dominant in AVG-MRSA. Of the 41 MSSA isolates, 10 STs were identified in 28 isolates before the pandemic, and 8 STs in 13 isolates during the pandemic (Figure 2B). ST15 was the predominant ST both before (28.6%) and during (30.8%) the pandemic. Before the pandemic, ST15 was prominent in AVG-MSSA, whereas ST188 was dominant in TCC-MSSA. The distribution of ST changed during the pandemic, as particularly exhibited by the decrease in AVG-MSSA ST15, the absence of AVG-MSSA ST188, the considerable increase in TCC-MSSA ST15, and the presence of TCC-MSSA ST97.

### 2.4. Prevalence of Antibiotic Resistance in VAI Isolates

**Figure 3:**
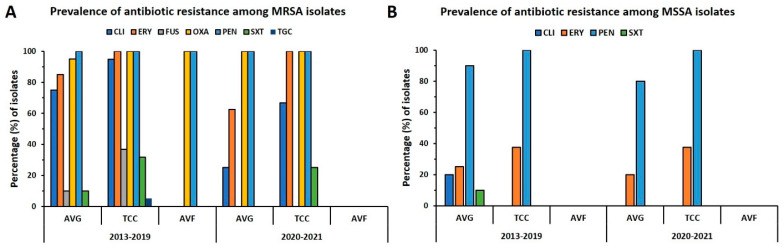
Distribution of antibiotic resistance in *S. aureus* isolates from VAIs before and during the pandemic (2013–2019 vs. 2020–2021). Resistance rates to clindamycin (CLI), erythromycin (ERY), fusidic acid (FUS), oxacillin (OXA), penicillin (PEN), trimethoprim-sulfamethoxazole (SXT), and tigecycline (TGC) in (**A**) MRSA and (**B**) MSSA isolates in the two periods by vascular access type.

The distribution of antibiotic resistance in MRSA and MSSA isolates from different types of VAIs is summarized in Figure 3. Each of the MRSA isolates was resistant to penicillin, and all but one was oxacillin-resistant. For the prepandemic period, more than 80% of isolates were resistant to clindamycin and erythromycin, wherein 75% of isolates for AVG-MRSA and 94.7% of isolates for TCC-MRSA were clindamycin-resistant and 85% of isolates for AVG-MRSA and 100% of isolates for TCC-MRSA were erythromycin-resistant, respectively. However, the rate of resistance to clindamycin was reduced to 50% during the pandemic, whereas the resistance rate was remarkably reduced to 25% for AVG-MRSA isolates and 66.7% for TCC-MRSA isolates. The rate of resistance to erythromycin for AVG-MRSA decreased to 62.5% during the pandemic; by contrast, the resistance rate of TCC-MRSA was the same as before. The rate of resistance to SXT decreased slightly during the pandemic, especially because none of the AVG-MRSA isolates were resistant to SXT. The MSSA isolates consistently exhibited high resistance to penicillin. The rate of resistance to penicillin and erythromycin in AVG-MSSA decreased slightly during the pandemic, and the AVG-MSSA antibiotic resistance rate was lower than TCC-MSSA. Overall, the AVG isolates generally exhibited lower resistance to the tested antibiotics than the TCC isolates, and this trend was more apparent during the pandemic.

### 2.5. Correlation of STs with Antibiotic Resistance

The antibiotic resistance profiles of each ST from different VAIs before and during the pandemic are summarized in Table 1. ST5, ST45, ST59, and ST239 were the STs mainly associated with clindamycin and erythromycin resistance in AVG-MRSA in the prepandemic period. An absence of ST5 and ST239 isolates and a loss of clindamycin and erythromycin resistance in ST45 isolates occurred during the pandemic, indicating that the change in ST distribution in AVG-MRSA during the pandemic led to a reduction in the rate of resistance to clindamycin and erythromycin. A similar phenomenon was observed for clindamycin resistance in TCC-MRSA during the pandemic, suggesting that the distribution of STs was considerably correlated with antibiotic resistance in isolates from different VAIs. The MSSA isolates with different STs were mainly resistant to penicillin and sometimes erythromycin; this was particularly true for ST7, ST12, ST15, and ST188. ST30 and ST239 were the AVG-MSSA isolates exhibiting clindamycin resistance; they exhibited similar resistance patterns to MRSA-ST30 and MRSA-ST239.

### 2.6. Correlation of Resistance Genotype with Phenotype

**Figure 4:**
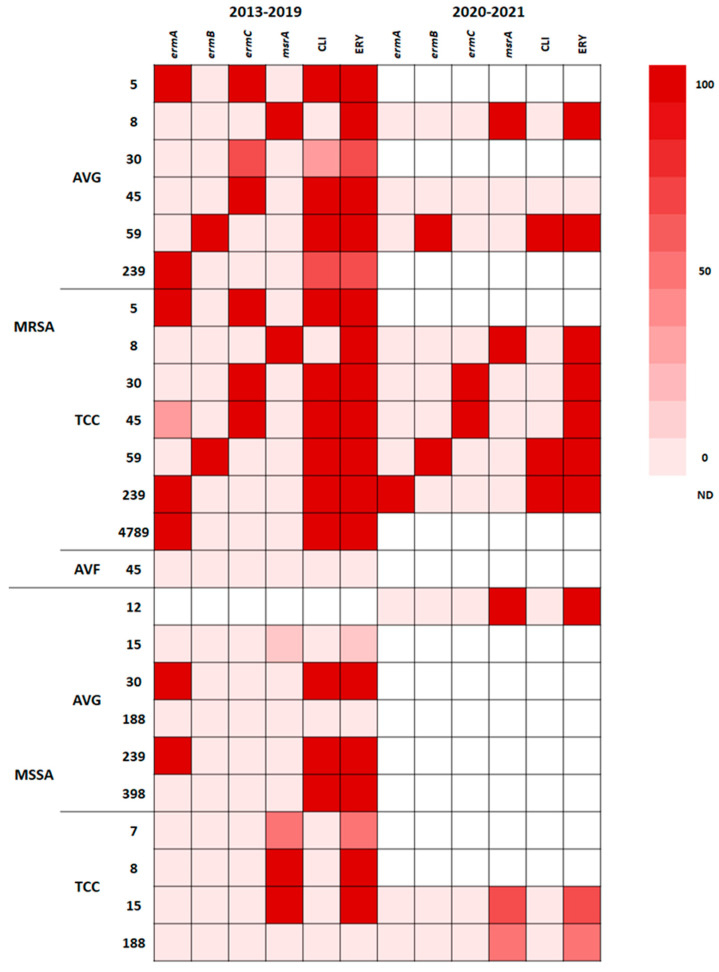
Heat map showing phenotypic and genotypic erythromycin resistance patterns in *S. aureus* isolates from VAIs.

Macrolide (erythromycin)–lincosamide (clindamycin)–streptogramin B resistance is typically mediated by a ribosomal RNA methylase encoded by erm genes through ribosomal target site methylation [15]. The phenotypic and genotypic resistance traits of isolates with different STs compared to erythromycin and clindamycin are presented in the heat map shown in Figure 4. The distribution of erm and msrA genes varied in different STs; 100% of the ST5 MRSA and ST59 MRSA isolates from AVG and TCC infections were resistant to clindamycin and erythromycin; ST5 MRSA harbored the *ermA* + *ermC* genes, whereas ST59 MRSA harbored the *ermB* gene. AVG-MRSA ST45 and TCC-MRSA ST45, predominantly carrying the *ermC* gene, were resistant to clindamycin and erythromycin, but one TCC-MRSA isolate carried the *ermA* + *ermC* genes; by contrast, AVF-MRSA ST45 was susceptible to clindamycin and erythromycin and carried no erm genes. MRSA and MSSA ST239 carrying the *ermA* gene exhibited clindamycin and erythromycin resistance; one AVG-MRSA ST239 isolate carried *ermA* but did not have clindamycin or erythromycin resistance, suggesting a mutation of the *ermA* gene. ST8 MRSA and MSSA isolates harboring the msrA gene were only resistant to erythromycin. The MSSA isolates of different STs that exhibited erythromycin resistance harbored the msrA gene. Both before and during the pandemic, the distribution of genotypic and phenotypic resistance to erythromycin and clindamycin of *S. aureus* was closely correlated with the ST.

## 3. Discussion

Because vascular access provides repeated access to the circulation, effectively functioning vascular access is crucial to efficient hemodialysis [16]. However, hemodialysis patients appear more vulnerable to *S. aureus* infection than others because vascular access can provide a route for *S. aureus* colonization and transmission; the type of vascular access is also associated with the specific risk of infection. In this single-institution study, *S. aureus* isolation rates differed considerably by the type of VAI, and the proportion of TCC infections increased and AVG infections decreased during the pandemic. The overall proportion of MRSA to MSSA was the same, approximately 3:2, before and during the pandemic. Nevertheless, the ratio of TCC-MRSA to TCC-MSSA declined during the pandemic; the ratio of AVG-MRSA to AVG-MSSA exhibited the opposite trend. The main reason for the increased use of TCCs during the pandemic was that timely maintenance of AVGs was difficult at this time. Once AVG dysfunction occurred, patients were more likely to be administered a TCC for hemodialysis than receive a thrombectomy of the AVG. Additionally, because TCC infections mostly occur in community-based clinics, their resistance to antibiotics is often greater than infections contracted in hospitals.

Regarding MLST, the clonal spread in hemodialysis patients and *S. aureus* infection was explored before and during the pandemic. In Taiwan, the community-associated ST59 became a major clone in hospital settings in the 2010s [17]. A surveillance study conducted across 18 provinces of China reported the dominance of ST59 between 2014 and 2019 [18], and Zhang et al. reported the dominance of ST59 in MRSA isolates in Anhui Province, China, in the 2020s [19]. Nevertheless, a study performed from 2009 to 2014 in a medical center in Southern Taiwan revealed that ST239 was the most common MLST type in hemodialysis cases (23.9%), followed by ST59 (17.7%) and ST45 (13.5%); however, infections by community-associated genotypes are increasing in the hemodialysis population [11]. In the present study, ST59 was the most predominant ST in the VAI MRSA population before and even during the pandemic and was especially prevalent in TCC-MRSA during the pandemic. MRSA ST45 and ST239 were also predominant in the prepandemic period. ST45 has been reported as endemic in nursing homes and long-term care facilities in Taiwan [20,21] and is the second leading nasal MRSA colonization in emergency department patients and healthcare workers in central Taiwan [22]. Patients receiving hemodialysis that are frequently shuttled between dialysis centers (healthcare facilities) and hospitals for healthcare may be the inadvertent cause of the dissemination of ST59 and ST45 within communities and hospitals. ST8 supplanted ST45 to become the second most prevalent ST after ST59 during the pandemic. The community-associated ST8 was initially the dominant clone in the United States [23,24] but gradually spread worldwide; identification of it in Asia, including Taiwan, has not been uncommon since 2010 [25,26,27]. A multicenter MRSA surveillance study conducted between 1995 and 2015 in Taiwan reported that 85% of MRSA ST8 isolates were identified after 2010, with their first identification being in 2005 [27]. A study conducted between 2016 and 2018 in northern Taiwan indicated that after an abrupt increase in prevalence, ST8 became the most prevalent ST in 2018, even replacing ST59 in community-associated settings [28]. However, the study indicated a low proportion of ST8 in catheter- and device-related infections; this may explain why ST8 was undetected in VAIs before the pandemic. Whether the high prevalence of ST8 during the pandemic was due to clonal expansion from community to hospital settings and high fitness in the hemodialysis population requires prolonged observation.

Exploring the contribution of antibiotic resistance over time is essential if appropriate drugs are to be selected for treating infections and the stockpiling of resistant bacteria is to be reduced. The prevalence of antibiotic resistance patterns is highly associated with STs. The overwhelming majority of *S. aureus* isolates (~97%) from VAIs were resistant to β-lactam penicillin, and approximately 60% of the isolates were oxacillin-resistant. Before the pandemic, TCC-MRSA was highly resistant to clindamycin (94.7%) and erythromycin (100%), in addition to penicillin and oxacillin, and exhibited a remarkably higher resistance rate than AVG-MRSA (75% and 85%, respectively). Although the rate of resistance to clindamycin was reduced during the pandemic, TCC-MRSA continued to exhibit higher resistance than AVG-MRSA. ST59 and ST239 isolates exhibit high rates of resistance to clindamycin and erythromycin [17,29], which is consistent with the current study’s finding that all ST59 and most ST239 isolates were resistant to both of these antibiotics. Consequently, the prevalence of ST59 and ST239 in TCC-MRSA during the pandemic led to higher clindamycin and erythromycin resistance rates than AVG-MRSA. The increase in the clindamycin susceptibility rate in Taiwan is mainly due to the increase in ST8 prevalence [30]. The increasing rate of ST8 during the pandemic directly reflected the decline in the clindamycin resistance rate because none of the ST8 exhibited clindamycin resistance. Other studies have indicated that the clindamycin resistance rate of ST8 was 7.7%, 66.7%, and 44.6% in northern Taiwan between 2016 and 2018 [28]; in Anhui Province, China, between 2021 and 2022 [19]; and in Japan in 2019 [31], respectively, suggesting geographical variation depending on local antibiotic usage or different genetic distributions.

Both before and during the pandemic, all ST8 isolates, including MRSA and MSSA isolates, harboring the msrA gene exclusively exhibited erythromycin resistance because clindamycin is neither an inducer nor a substrate for the msrA-mediated efflux pump, which is responsible for pumping macrolide and streptogramin B antibiotics out of bacteria [32]. Nevertheless, a study conducted in Japan reported a high rate of retention of the *ermA* gene in ST8 isolates with almost the same clindamycin resistance rate [31], suggesting that the spread of resistance genes is possibly confined geographically. Wang et al. [33] reported that MRSA ST59 isolates from children in northern Taiwan between 1997 to 2002 were resistant to erythromycin and clindamycin and had the *ermB* gene, which is similar to our finding. A study conducted in Hangzhou, China [34], demonstrated that ST59 predominantly carried the *ermB* gene as dominant and, to a lesser extent, the *ermC* gene, wherein the transmissible *ermC* gene was also exhibited in other STs. In our study, the *ermC* gene was detected in AVG-MRSA and TCC-MRSA ST5, ST30, and ST45 with clindamycin and erythromycin resistance before the pandemic but in TCC-MRSA ST30 and ST45 with erythromycin resistance during the pandemic, suggesting that the management of antibiotic usage or clonal contraction may confine the spread of resistance genes.

This study provided insight into molecular characteristics and phenotypic and genotypic antibiotic resistance in *S. aureus* isolates from different types of VAIs before and during the pandemic. Nonetheless, this study has several limitations. This was a single-institution longitudinal study covering an 8-year period; the varying frequency of isolates collected each year and the small sample size may have caused bias; however, the data nonetheless reflect the situation during this study. Furthermore, because of the lack of patients’ demographic data, the impact of changes in STs on the clinical course is unclear. Although our findings may not represent the different STs circulating in the hemodialysis population with *S. aureus* VAIs in different geographic regions and periods, the data provide insight into the distribution of STs and the spreading of antibiotic resistance in infection control and management.

## 4. Materials and Methods

### 4.1. Ethical Approval

This study was approved by the Institutional Review Board (IRB) of Chang Gung Memorial Hospital (IRB201508482B and IRB201901354B0).

### 4.2. Study Setting, Bacterial Isolate Collection, and Identification

The study was conducted at a tertiary teaching hospital, Chiayi Chang Gung Memorial Hospital in Chiayi, Taiwan, between November 2013 and December 2021. A total of 102 bacterial isolates were collected from hemodialysis patients for whom infected TCCs, AVGs, and AVFs had to be removed. The bacterial isolates were derived from contaminated Hickman catheter tips, wounds, pus, abscesses, and blood, and were cultured on the blood agar plate (BAP) by the Department of Laboratory Medicine. Before 2019, the isolates were identified using standard biochemical tests, including the catalase and coagulase tests, and matrix-assisted laser desorption/ionization time-of-flight mass spectrometry (MALDI-TOF) was used after 2019. The isolates were routinely cultured on tryptic soy agar and tryptic soy broth under laboratory standards. All isolates were frozen in a 15% glycerol stock and kept at −80 °C.

### 4.3. Antimicrobial Susceptibility Testing

The antimicrobial susceptibility of *S. aureus* isolates was determined using disk diffusion with the following antibiotics: clindamycin, erythromycin, fusidic acid, oxacillin, penicillin, trimethoprim-sulfamethoxazole (SXT), and tigecycline. The results were interpreted in accordance with the standards of the Clinical and Laboratory Standards Institute [35].

### 4.4. Molecular Characterization and Antibiotic-Resistant Gene Detection

#### 4.4.1. Identification of Methicillin-Resistant *S. aureus* and Staphylococcal Chromosomal Cassette mec (SCCmec) Type

Genomic DNA was extracted using a method previously described [36]. The isolates were identified as MRSA when they exhibited oxacillin resistance and *mecA* positivity. The detection of *mecA* was performed using a polymerase chain reaction (PCR) with previously described primer pairs [37]. This study also categorized the oxacillin-resistant *mecA*-negative and oxacillin-sensitive *mecA*-positive isolates as MRSA. SCC*mec* types I–V were identified using a multiplex PCR assay together with specific primers [38].

#### 4.4.2. Molecular Typing

For the 102 isolates, multilocus sequence typing (MLST) was performed by amplifying the internal fragment of 7 housekeeping genes through a previously described protocol followed by sequencing [39]. When the *aroE* gene could not be amplified, we used alternative primers described by Schuster et al. [40]. The amplified product sequencing in both directions was performed using Sanger dideoxy DNA sequencing (Mission Biotech, Taipei, Taiwan). The sequence type (ST) of each isolate was determined using BioNumerics 7.6 (Applied Maths, Sint-Martens-Latem, Belgium) in accordance with the MLST database [41].

#### 4.4.3. Erythromycin-Resistant Gene Detection

Several antibiotic-resistant genes were identified using multiplex PCR with the 16S rDNA gene as an internal control. The following genes conferring resistance to erythromycin were screened: *ermA*, *ermB*, *ermC*, and *msrA* [42].

## 5. Conclusions

During the pandemic, AVG-related infections decreased, and TCC-related infections increased. The prevalence of various STs differed by VAI type and changed from before the pandemic to during the pandemic. ST8 took over from ST59 as the dominant ST in AVG-MRSA, and ST59 increased remarkably in TCC-MRSA during the pandemic. The change in antibiotic resistance rate in different VAIs between the two periods was closely related to the distribution of STs because some STs carried specific resistance genes. The molecular surveillance of *S. aureus* VAIs is crucial in tracing the expansion/reduction in certain clones for infection management and further delineating an effective therapeutic strategy.

## Figures and Tables

**Table 1:** Antibiotic resistance pattern in MRSA and MSSA isolates on the basis of STs before and during the pandemic.

ST	2013–2019			2020–2021		
	AVG	TCC	AVF	AVG	TCC	AVF
MRSA						
5	CLI, ERY, OXA, PEN (1)	CLI, ERY, FUS, OXA, PEN (1)				
8	ERY, OXA, PEN (1)	ERY, OXA, PEN (1)		ERY, OXA, PEN (3)	ERY, OXA, PEN (2)	
30	CLI, ERY, OXA, PEN (1)	CLI, ERY, OXA, PEN (2)			ERY, OXA, PEN (1)	
	ERY, OXA, PEN (1)					
	OXA, PEN (1)					
45	CLI, ERY, FUS, OXA, PEN (2)	CLI, ERY, FUS, OXA, PEN (3)	OXA, PEN (2)	OXA, PEN (2)	ERY, OXA, PEN (1)	
	CLI, ERY, OXA, PEN (2)					
59	CLI, ERY, OXA, PEN (7)	CLI, ERY, OXA, PEN (6)		CLI, ERY, OXA, PEN (2)	CLI, ERY, OXA, PEN (5)	
72				OXA, PEN (1)		
239	CLI, ERY, OXA, PEN, SXT (2)	CLI, ERY, FUS, OXA, PEN, SXT (2)			CLI, ERY, OXA, PEN, SXT (3)	
	PEN (1)	CLI, ERY, OXA, PEN, SXT (2)				
		CLI, ERY, OXA, PEN, SXT, TGC (1)				
508	OXA, PEN (1)					
4789		CLI, ERY, FUS, OXA, PEN, SXT (1)				
MSSA						
1		PEN (1)				
6				PEN (1)		
7	PEN (2)	ERY, PEN (1)				
		PEN (1)				
8	PEN (2)	ERY, PEN (1)				
12				ERY, PEN (1)		
15	ERY, PEN (1)	ERY, PEN (1)		PEN (1)	ERY, PEN (2)	
	PEN (6)				PEN (1)	
30	CLI, ERY, PEN (1)					
96				PEN (1)		
97	PEN (1)				PEN (2)	
188	PEN (1)	PEN (3)			ERY, PEN (1)	
	NONE (1)				PEN (1)	
239	CLI, ERY, PEN, SXT (2)					
398	CLI, ERY (1)					
845	PEN (2)					
2260				NONE (1)		
6892					PEN (1)	

Note: AVG: arteriovenous graft; TCCs: tunneled cuffed catheters; AVF: arteriovenous fistula; MRSA: methicillin-resistant *S. aureus*; MSSA: methicillin-sensitive *S. aureus*; CLI: clindamycin; ERY: erythromycin; OXA: oxacillin; PEN: penicillin; FUS: fusidic acid; SXT: trimethoprim-sulfamethoxazole; TGC: tigecycline; ST: sequence type.

## Data Availability

The authors declare that the experimental data published in this paper are made accessible upon request for interested readers.
